# Cataloguing the dead: breathing new life into pseudokinase research

**DOI:** 10.1111/febs.15246

**Published:** 2020-03-10

**Authors:** Safal Shrestha, Dominic P. Byrne, John A. Harris, Natarajan Kannan, Patrick A. Eyers

**Affiliations:** ^1^ Institute of Bioinformatics University of Georgia Athens GA USA; ^2^ Department of Biochemistry & Molecular Biology University of Georgia Athens GA USA; ^3^ Department of Biochemistry Institute of Integrative Biology University of Liverpool Liverpool UK

**Keywords:** bioinformatics, Eph tyrosine kinase, ephrin receptor, inhibitor, pseudoenzyme, pseudokinase, PSKH1, PSKH2, sequence analysis, signalling, tribbles

## Abstract

Pseudoenzymes are present within many, but not all, known enzyme families and lack one or more conserved canonical amino acids that help define their catalytically active counterparts. Recent findings in the pseudokinase field confirm that evolutionary repurposing of the structurally defined bilobal protein kinase fold permits distinct biological functions to emerge, many of which rely on conformational switching, as opposed to canonical catalysis. In this analysis, we evaluate progress in evaluating several members of the ‘dark’ pseudokinome that are pertinent to help drive this expanding field. Initially, we discuss how adaptions in erythropoietin‐producing hepatocellular carcinoma (Eph) receptor tyrosine kinase domains resulted in two vertebrate pseudokinases, EphA10 and EphB6, in which co‐evolving sequences generate new motifs that are likely to be important for both nucleotide binding and catalysis‐independent signalling. Secondly, we discuss how conformationally flexible Tribbles pseudokinases, which have radiated in the complex vertebrates, control fundamental aspects of cell signalling that may be targetable with covalent small molecules. Finally, we show how species‐level adaptions in the duplicated canonical kinase protein serine kinase histone (PSKH)1 sequence have led to the appearance of the pseudokinase PSKH2, whose physiological role remains mysterious. In conclusion, we show how the patterns we discover are selectively conserved within specific pseudokinases, and that when they are modelled alongside closely related canonical kinases, many are found to be located in functionally important regions of the conserved kinase fold. Interrogation of these patterns will be useful for future evaluation of these, and other, members of the unstudied human kinome.

## Graphical abstract

Here, we evaluate several families of poorly studied 'dark' kinases and pseudokinases using a bioinformatic and structural analysis approach. Our investigation revealed several new features of two erythropoietin‐producing hepatocellular carinoma (Eph) psedudokinases: EphA10 and EphB6. Furthermore, we discuss newly identified features of the Tribbles pseudokinases as well as the unique pseudokinase protein serine kinase histone 2 (PSKH2).

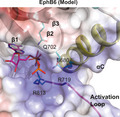

**Abbreviations**
C/EBP: CCATT‐enhancer‐binding protein
CAMK: calcium calmodulin kinase
CHA: contrast hierarchical alignment
Eph: erythropoietin‐producing hepatocellular carcinoma
JAK: Janus kinase
PKA: cAMP‐dependent protein kinase
PSKH: protein serine kinase histone
SAM: sterile alpha motif
STK: serine‐threonine kinase
TRIB: Tribbles
WNK: with no lysine

## Introduction

Protein kinases control all aspects of signalling throughout the different kingdoms of life 1. Bioinformatic kinome analysis reveals their evolutionary‐conserved division into canonical (catalytically active) kinases and pseudokinases, which are usually catalytically inactive 2.

Pseudokinases, in particular, and pseudoenzymes, in general, 3 have received only a fraction of the attention bestowed on their catalytically active relatives, despite widespread evidence in the kinase field that both kinases and pseudokinases 4 employ switch‐like transitions to co‐operatively regulate signalling networks in health and disease 5. Fundamental protein kinase research emerged rapidly during development of the cell signalling field in the late 1970s, driven first by biochemical (enzyme‐based) and then genetic (phenotypic) studies 6. The realization that protein kinases are druggable targets in disease, combined with pharmaceutical buy‐in, led to rapid breakthroughs of therapeutic importance in the early 2000s 7, and as of 2020, some 65 kinase inhibitors that target canonical kinase domains have been approved. In contrast, and despite their initial analysis two decades ago by Manning and colleagues, 8, 9 conventional challenges facing researchers seeking to understand pseudokinase biology through hypothesis‐driven approaches are compounded by a lack of tools (e.g. small molecules) and technologies (e.g. biophysical and cellular biomarkers) with which to evaluate mechanistic and cellular functions 10. Despite this, progress has recently been made in some areas of ‘dark’ kinome investigations 11, including evaluation of the associated ‘dark’ phosphoproteome 12. Indeed, new open‐access collaborative efforts between academia and pharma have generated potential breakthroughs of relevance for pseudokinases whilst driving widespread recognition of catalytically independent functions of canonical kinases 13 and pseudokinases 14. Excitingly, the ‘Illuminating the Druggable Genome’ programme, which began as a US National Institutes of Health Common Fund pilot project in 2014, has now been implemented to accelerate ‘the investigation of subsets of understudied proteins that have potential therapeutic relevance’ 15, 16, of which pseudokinases (and other pseudoenzymes) form an important class. However, it is clear that evaluating the mechanistic outputs of all human pseudokinases, and establishing their biological functions and respective cellular niches, remains a major research goal. For example, although approximately half of human pseudokinases are still able to bind to nucleotides, the functional consequences of this event remain obscure in nearly all cases, perhaps due to a current lack of cellular reporters designed to report pseudokinase conformations 17. Ever‐closer collaboration between specialists in various fields fully supports the notion that molecular dissection of pseudokinase‐based signalling mechanisms will be enhanced if the decades of combined experience focused on analysing (canonical) protein kinases can be concentrated and then refocused. In this review, we highlight such an approach by employing comparative data mining and structure‐based modelling to compare and contrast current understanding of three small families of relatively poorly studied members of the human kinome, the Tyr pseudokinases erythropoietin‐producing hepatocellular carcinoma (Eph)A10 and EphB6, Tribbles/TRIB/STK40 and the orphan pseudokinase protein serine kinase histone (PSKH)2, whose biological function still remains unknown despite clear conservation in most vertebrates.

## Evaluating the underexplored and ‘dark’ pseudokinome

### Eph tyrosine kinases and pseudokinases

The ephrin (Eph) subfamily of transmembrane‐containing receptors represents the most abundant grouping of receptor tyrosine (Tyr) kinases in humans and is intimately linked to cell‐based proliferative diseases, including cancer 18. However, in contrast to Tyr kinases that have been targeted intracellularly with small molecules and extracellularly with therapeutic antibodies, the development of drugs to modulate Eph receptor function remains in its infancy, despite a reasonable (but by no‐means complete) level of understanding of their molecular signalling mechanisms. In this context, complexity amongst the members of the Eph family is underscored by the eukaryotic conservation of two pseudokinases, EphA10 and EphB6, whose functions remain poorly characterized despite recent advances in understanding their broader signalling roles 19, 20. Structural data suggest that canonical tyrosine kinase‐containing Eph receptors are subject to complex regulatory coupling to ephrin ligand occupancy and intracellular protein:protein interactions, some of which might represent kinase‐independent functions in canonical Eph tyrosine kinase domains 21 and some of which might feasibly be driven by conformational changes in the pseudokinase domains of EphA10 and EphB6 19. Eph receptors are characterized by the presence of either a GPI‐linked receptor binding domain or a multidomain extracellular ephrin‐binding domain, whose differential affinity for ephrin ligands and clustering abilities 22, 23, 24 are transduced across the membrane via intracellular tyrosine kinase‐based mechanisms. Inside the cell, a transmembrane helical region, a short juxtamembrane (JM) region, which is inhibitory to catalysis until Tyr phosphorylated in response to ligand 25, and an intracellular kinase domain are all coupled to a C‐terminal sterile α‐motif (SAM) domain that terminates with a docking motif for PDZ domain‐contain proteins termed the PBM. In terms of signalling outputs, the JM domain maintains the kinase domain in an inactive conformation and can be modulated by phosphorylation of two highly conserved Tyr residues (see below). It also serves as a Tyr‐phosphorylated platform for recruitment of partner proteins through SH2 domain‐based interactions. C‐terminal to the Eph (pseudo)kinase domain, a short linker region, leads to the SAM domain, an oligomerization domain for higher‐order assembly of complexes that control outputs from the kinase domain. The SAM domain linker is allosterically coupled to the JM domain via conserved residues in the EphA3 kinase domain 26.

### EphA10 and EphB6 form a pseudokinase ‘subfamily’

Pseudokinase domain sequence comparisons between EphA10 and EphB6 argue (but does not prove) that these catalytically inactive pseudokinases initially emerged and then became specialized, through gene duplication events from catalytically competent homologues, EphA7 in the case of EphA10 and EphB1 in the case of EphB6 20. When assessed side‐by‐side, the pseudokinase domains of EphA10 and EphB6 possess an overall amino acid identity of ~ 50%, with a similarity of ~ 65%. In terms of their intracellular regions, major similarities between EphA10 and EphB6 include the absence of all three catalytic residues in the pseudokinase domain, and this can be readily appreciated through comparative amino acid analysis 20 and inspection of Table 1. An example of specific differences is the JM region of EphA10, which lacks phosphorylatable Tyr residues (termed JX1 and JX2 in EphB6, where they are conserved, 18), but the EphA10 activation segment (following the degraded ‘DFG’ motif) contains a canonical Tyr in the ‘T‐loop’ site, a classical site of activating phosphorylation, whereas the activation segment of EphB6 is quite divergent (Tables 1 and 2). Neither EphB6 nor EphA10 would be predicted *a priori* to be catalytically active, since they lack all three canonical motifs corresponding to the VAIK, DFG and extended HRD sequences, which function to position nucleotide and cations and catalyse phosphate transfer to substrate in enzymatically active kinases 27.

**Table 1:** Key catalytic and structural motifs in the pseudokinase domain*.* Human Eph, TRIB and PSKH1/2 motif residues, including many discussed in the text, are depicted in single amino acid code. Residues believed to be critical for nucleotide and metal binding, positioning and catalysis are underlined

Name of region in domain	Canonical sequence 27	EphA10	EphB6	PSKH1	PSKH2
Gly‐rich region (P‐loop)	GXGXXG	GGGRFG	GTGSFG	GRGSFS	GTGSFS
β2 small C‐spine residue	V/A/G	L	V	V	V
β3 strand motif XAXK	VAIK	VAVH	VAIQ	YAIK	FAIK
Gatekeeper residue	M/T/L/F/V	T	T	M	M
C‐helix Glu	E	E	R	E	E
Catalytic loop	HRDXXXXN	HRGLAARH	HRSLSAHS	HRDLKPEN	HRNLKPEN
DFG motif	DFG	GFG	RLG	DFG	DFG
Activation segment residues	Y, S or T	Y	‐	T	T
F‐helix Asp	D	D	D	D	D

Name of region in domain	Canonical sequence 27	TRIB1	TRIB2	TRIB3	STK40
Gly‐rich loop (P‐loop)	GXGXXG	EREHVS	EGDHVF	EGGRAY	GNSPVP
β2 small C‐spine residue	V/A/G	A	A	A	Q
β3 strand motif	VAIK	LRCK	LVCK	YTCK	YQLK
Gatekeeper residue	M/T/L/F/V	F	F	F	F
C‐helix Glu	E	D	E	A	E
Catalytic loop	HRDXXXXN	LGDLKLRK	LRDLKLRK	LRDLKLCR	HRDLKLGN
DFG motif	DFG	ESLED	ESLED	ENLED	TNFCL
Activation segment residues	Y, S or T	S, C	S, C	S, C	S
F‐helix Asp	D	D	D	D	D

**Table 2:** Reported phosphorylation sites in vertebrate pseudokinases. Phosphorylation site data were harvested from PhosphoSitePlus™ version 6.5.8 (phosphosite.org, Jan 2020), and conserved sites of phosphorylation of interest identified by shotgun phosphoproteomics are shown, with human numbering and position in the pseudokinase noted where appropriate. For several of these sites, notably the juxtamembrane phosphorylation sites in EphB6 (several of which are absent in EphA10), experimental evidence has confirmed the potential importance of Ser, Thr or Tyr phosphorylation. It is likely that focused analysis of these pseudokinases in different species and cell types, and under different experimental conditions, will reveal additional phosphorylation sites. In most cases, further experimentation is also needed to confirm phosphorylation and to identify potential ‘upstream’ kinases in each case

Pseudokinase	UniProt ID (human)	Amino acids (pseudokinase domain)	Phosphorylation sites conserved between human and murine sequences
EphA10	Q5JZY3	645–900	Ser 756 (αE)/Ser 779 (between catalytic and activation loops). Ser 805/Ser 808 in the activation segment not currently known to be phosphorylated
EphB6	O15197	670–919	Tyr 628/Tyr 635 (juxtamembrane), Tyr 644, Tyr655 (JX1), Tyr 651 (JX2), Tyr 669, Tyr 785 (conserved in all Eph kinases)
TRIB1	Q96RU8	84–343	Ser 8 (PEST domain), Thr 229 (end of SLE motif)
TRIB2	Q92519	61–308	Tyr 14 (PEST domain), Ser 83 (p70S6k), Ser 133/Tyr 134 (hinge region), Thr 143, Tyr 218, Thr 276, Ser 278, Thr 298/Ser 299
TRIB3	Q96RU7	68–316	Ser 14 (PEST domain), Ser 51 (SP site), Thr 64, Ser 215
STK40/Sgk495	Q8N2I9	35–331	Ser 95, αC‐helix adjacent to Glu 93
PSKH1	P11801	98–355	Tyr 35, Thr 131 (end of β3), Thr 164/Thr 176
PSKH2	Q96QS6	63–320	Tyr 63, Thr 84/Thr 85 (between β2 and β3), T‐loop Thr 221, Ser 348 (no evidence currently), Ser 356/Ser 359/Ser 362 C‐term cluster identified

### An experimentally validated nucleotide‐binding fold in EphB6

Interestingly, the ‘glycine‐rich region’ of the P‐loop is essentially intact in both EphB6 and EphA10 (Table 1), in line with experimental demonstration of nucleotide (in the absence of divalent metals *in vitro*) and kinase inhibitor binding for EphB6 28, 29, 30. To our knowledge, however, the ability of EphA10 to bind to nucleotides has not been confirmed, or even evaluated, experimentally. Moreover, biochemical analysis of R813D EphB6, in which a canonical Asp residue replaces the charged Arg at the cryptic ‘DFG’ motif, reveals broad inhibitory effects on nucleotide binding, suggesting an atypical Arg‐dependent mode for metal‐independent ATP and GTP binding 28, which highlights the need for a broader analysis of the residue composition of Eph pseudokinases. Interestingly, the binding of several kinase inhibitors to EphB6 analysed by differential scanning fluorimetry is independent of Arg813 28 and suggestive of a strong (potentially nM) interaction *in vitro*. This finding is consistent with cellular competition data 30 and predicts the presence of a cavity through which relatively planar molecules, such as type I ATP‐competitive inhibitors, can target EphB6. This notion is discussed in more detail below.

### Ephrin receptor residue conservation and structural analysis

We took advantage of the thousands of Eph kinase and pseudokinase sequences in sequence databases and implemented evolutionary sequence and structure‐based analysis to model dynamic, likely nucleotide‐dependent (but kinase‐independent) signalling properties of these enigmatic pseudokinases (Figs S1 and S2). We used the optional multiple‐category Bayesian Partitioning with Pattern Selection (OmcBPPS) algorithm, a Markov chain Monte Carlo sampling method 31. This takes as its input multiple‐sequence pseudokinase alignments, creating and optimizing a hierarchy based on a probability distribution that models the conserved and divergent patterns defining each subgroup of pseudokinase. It also identifies distinguishing pattern residues for each of the subgroups and generates a statistical measure of divergence at specific residue positions. We previously applied these methods to evaluate other (pseudo)kinase subfamilies 26, 32, 33, and for the sequence alignment outputs, we have included a conventional kinase counterpart sequence to allow simple comparisons.

The absence of structural information for EphA10 and EphB6 is balanced by a large amount of structural information for closely related canonical Eph kinase domains, which have been crystallized in ‘active‐like’ (closed) and ‘inactive‐like’ (open) enzymatic conformations 34 that are ideal for modelling purposes 35, 36. This is in addition to an evolving appreciation of the cellular mechanisms by which catalytic output from Eph kinase and pseudokinase complexes are potentially coordinated 20, 24, 37, 38.

### EphA10‐specific amino acid divergence in the ‘nucleotide‐binding’ pocket

**Fig. 1:**
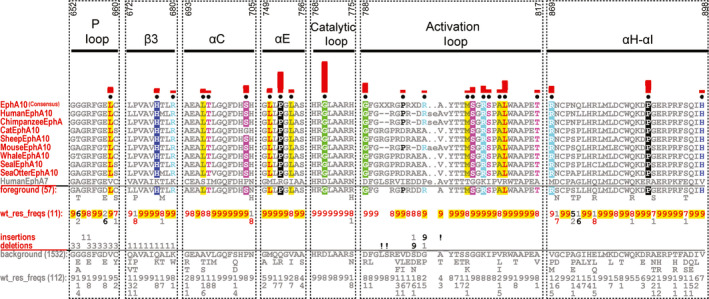
Analysis of EphA10 pseudokinase domain sequences. Sequence constraints that distinguish EphA10 from other Eph sequences are shown as a CHA 31. The CHA shows selected EphA10 sequences from diverse organisms as the display alignment, a foreground alignment of 57 EphA10 sequences and a background alignment of 1532 sequences. The foreground and background alignments are shown as residue frequencies below the display alignment in integer tenths (1–9). The histogram (in red) above the display alignment indicates the extent to which the distinguishing residues in the foreground alignment diverge from the corresponding position in the background alignment. Black dots mark the alignment positions used by the BPPS procedure 31 when classifying EphA10 from other Eph sequences. Alignment numbering (top) is based on the human EphA10 sequence, with the human EphA7 sequence provided for comparison. UniProt accession numbers for the sequence analysis are as follows: Human EphA10: Q5JZY3; Chimpanzee EphA10: H2PYP7; Cat EphA10: A0A212UHA0; Sheep EphA10: W5QF15; Mouse EphA10: Q8BYG9; Whale EphA10: A0A384A623; Seal EphA10: A0A2U3Y845; Sea Otter EphA10: A0A2Y9IWK0. Human EphA7: Q15375.

**Fig. 2:**
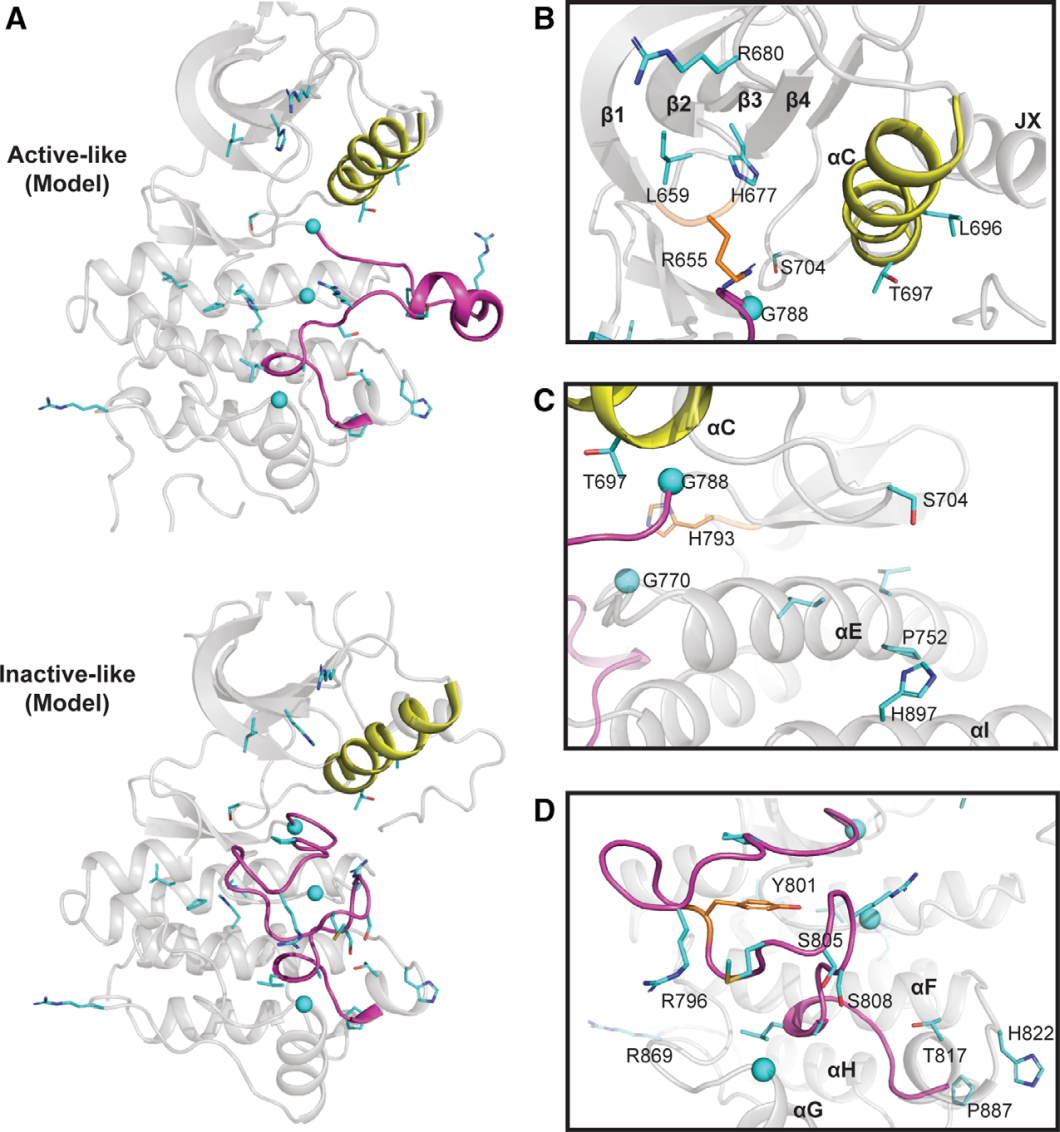
EphA10 homology models in ‘active‐like’ and ‘inactive‐like’ conformations. (A) Cartoon representation of the homology models of human EphA10 in the active EphA3 conformation (PDB ID: 3FXX) 39 and an inactive‐like EphA7 conformation (PDB ID: 3DKO). We used SWISS‐MODEL server 84 to find the best template, and the conformers are based on the template selected. EphA10‐specific pattern residues are shown as sticks (Gly residues as spheres) and coloured in cyan. The residues were identified using a BPPS procedure as described in the legend to Fig. 1. (B) EphA10‐specific divergence in the ‘nucleotide‐binding’ pocket. EphA10‐conserved Gly‐rich (P) loop residue Arg655 is coloured in orange and shown as sticks. (C) EphA10‐specific divergence in the E‐ and I‐helix face of the pseudokinase domain. (D) EphA10‐specific divergence in the activation loop. Zoomed‐in view of the activation loop in the ‘inactive‐like’ conformation is shown. Distinguishing residues are coloured in cyan and shown as sticks. C‐helix and activation loop are coloured in yellow and magenta, respectively, in panels A‐D. Molecular visualization was performed with pymol Molecular Graphics System, version 2.0 Schrodinger, Portland, OR, USA.

**Fig. 3:**
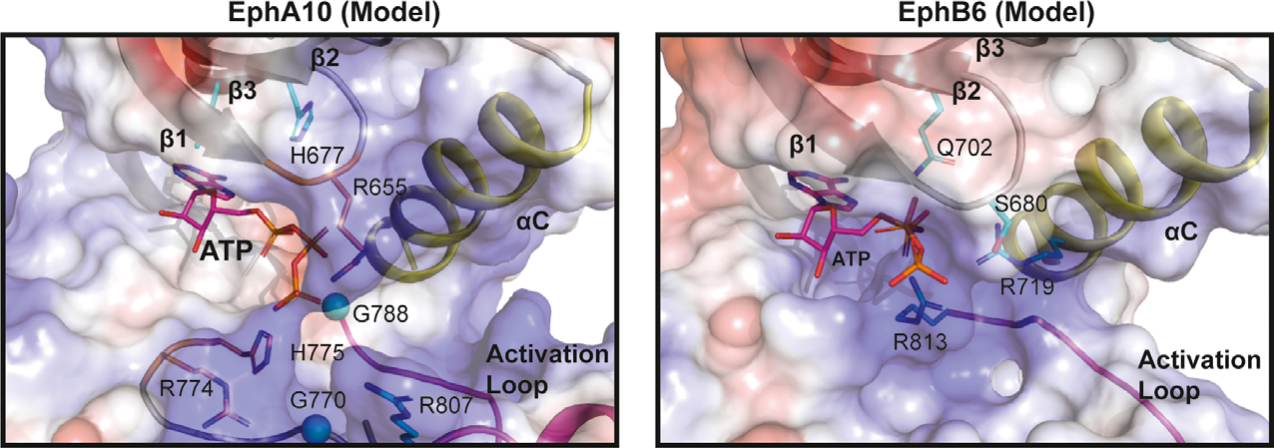
Surface electrostatics and ATP pose modelling in EphA10 and EphB6 ‘active‐like’ conformations. An ATP molecule, shown in sticks, was modelled in the absence of metal ions by aligning the structure of murine PKA (PDB ID: 1ATP) 85 to either EphA10 (left) or EphB6 (right) pseudokinases, which were modelled in an ‘active‐like’ closed conformation, using pymol 2.0. The ATP pose was further refined manually in pymol. EphA10‐ and EphB6‐specific residues are shown as sticks and coloured in cyan with glycine residues shown as spheres. EphA10‐conserved residues that are not part of distinguishing features from the Bayesian analysis are shown as sticks and coloured in orange. Positive patches on the surface are coloured in blue, whilst negatively charged surfaces are coloured in red. Hydrophobic surfaces are coloured in white. The αC and activation loops are labelled, as are the positions of residues of potential relevance to ATP binding.

Structural prediction and comparison of EphA10 with all other Eph family sequences confirms the divergence of a series of amino acids in the putative ATP‐binding pocket, alongside specialized residue conservation in the wider pseudokinase domain, which readily distinguish it from canonical EphA7 (Fig. 1). We also modelled EphA10 in both ‘active‐like’ (or closed) and ‘inactive‐like’ (or open) conformations (Fig. 2), based on the adoption of similar poses in catalytically competent EphA3 (PDB ID: 3FXX) 39 and ‘DFG‐out’ EphA7 complexed to ALW‐II‐49‐7 (PDB ID: 3DKO) 40, respectively. We initially speculated that EphA10 may have lost the ability to bind to nucleotides. In support of this, specific residues that might prevent such interactions include Leu659 and His677, which protrude from the β2 and β3 strands, respectively (Fig. 2A,B). Moreover, the presence of a larger hydrophobic Leu residue in place of Val, found in other Eph family kinase domains (including nucleotide‐binding proficient EphB6), might also alter the shape of the ATP‐binding pocket and, thus, alter the affinity for ATP binding. However, depending upon the molecular environment, and given the strong conservation of Gly residues in the EphA10 Gly‐rich loop (Table 1), the absence of bulkier amino acids that preclude binding of the purine ring in other pseudokinases, such as vaccinia‐related kinase 3 41 and STK40 (see below), and the conservation of Arg655 (Figs 1 and 2B) suggest that EphA10 has likely retained nucleotide binding. Indeed, the canonical positively charged β3 Lys is substituted with a His in EphA10 (Figs 1 and 3) as part of a ‘VAVH’ motif, where Ala at the second position might also contribute to nucleotide binding. Similar changes of the canonical Lys to Arg (in kinase suppressor of ras1/2) or Cys [in the catalytically active with no lysine (WNK) kinases] have been noted in the literature, although the Ala residue is conserved in both 27. A His residue at this position, which can be positively charged, neutral or form directional hydrogen bonds, could form a credible functional replacement for the canonical Lys, and if atypical nucleotide binding is relevant for EphA10, it is likely that this His residue will be required functionally. Analysis of the predicted electrostatic surface of EphA10 alongside EphB6 (Fig. 3) further supports this hypothesis, with the phosphate groups of ATP positioned facing towards a basic surface. Careful biochemical and biophysical experimentation with appropriately purified proteins will be needed to confirm the nucleotide‐binding potential for these pseudokinases.

### EphA10‐specific divergence in the substrate‐binding pocket

Our EphA10 homology model demonstrates several specific adaptions in the pseudokinase domain (Fig. 2). Firstly, and in the context of the N‐terminal JX (specifies phosphorylation sites on the juxtamembrane) region 26, we note that although the two JX Tyr residues are swapped for Phe and Cys, the positions of two αC‐helix residues, Leu696 and Thr697 (Fig. 2B), directly correspond to residues that form interactions with the juxtamembrane region in canonical Eph crystal structures such as EphA3 (PDB ID: 2QO2) 36. The replacement of comparatively smaller residues such as Ala, Ser or Thr with Leu (e.g. Leu 696) suggests that these substitutions could increase hydrophobic van der Waals contacts and stabilize interactions with the juxtamembrane region. On the other hand, a hydrophobic residue is present in other Eph family members in place of Thr697. The hydrogen bonding ability of Thr or an ability to become phosphorylated might potentially alter allosteric interactions between the αC‐helix and the JX region. Since EphA10 does not have the two phosphorylatable JX Tyr residues, these adaptions in the αC‐helix (Fig. 2B) might be employed to tether the JX membrane in a unique pseudokinase conformation involved in signal transmission (PDB ID: 2QO2). In addition, we note that EphA10 αC‐β4 loop residue Ser704 is present in place of a Pro residue, found in all Eph kinases except EphA2, where a His is present. Introduction of the hydrophilic (and phosphorylatable) Ser could suggest a potential new regulatory function for this residue. We also predict that introduction of Pro (Pro752) in the middle of the long αE‐helix might induce a ‘kink’ in the helix, which could subsequently change the spatial location of the adjacent *C*‐terminal residues (Fig. 2C). The presence of an EphA10‐specific histidine (His897) in the αI‐helix in place of the canonical hydrophobic Val residue could lead to the formation of regulatory hydrogen bonds with residues in the αE‐helix. Interestingly, Ser756, which is *C*‐terminal of Pro752, has been annotated as a phosphorylation site (Table 2). Ser756 is in proximity to His897, suggesting that a phosphorylation event might be stabilized by these EphA10‐specific residues. Finally, the invariant catalytic loop Asp of canonical kinases in the ‘HRD’ motif is replaced by Gly770 (Table 1, Fig. 1), strongly supporting the idea that the EphA10 subfamily has lost the ability to catalyse the phosphotransfer reaction. Moreover, an evolutionary‐conserved glycine (Gly788) is present in place of the magnesium‐binding Asp of the ‘DFG’ motif, alongside replacement of the canonical Asn involved in divalent metal ion binding, which is replaced by His793. Together, these changes confirm that the enzyme has lost the ability to bind to magnesium ions, which might be expected to be important for physiological stabilization of ATP but is unlikely to preclude interaction with small molecule ligands.

### EphA10 divergence in the activation segment

EphA10 contains an activation segment region with some similarities to canonical Eph kinase domains, whose conformation is controlled through phosphorylation (Fig. 2D). Quantitative comparison with other Eph family sequences also reveals key divergence in activation segment residues (Table 1). Two serine residues, Ser805 and Ser808, have been conserved in the EphA10 activation loop, where they replace highly conserved Gly and Ile residues found in other Eph kinase domains (Fig. 2D). This change not only introduces potential new phosphorylation sites for ‘upstream’ Ser/Thr kinases (Table 2) but would likely affect the dynamics of the unique EphA10 activation loop. Finally, there is an additional positively charged Arg (Arg869) present in the αH‐αI loop and a Pro (Pro887) in the loop connecting the αH‐helix and the αI‐helix (Fig. 2D). These EphA10‐specific residues might stabilize the SAM linker regions in a specific conformation, although a structure of the EphA10 pseudokinase domain and its corresponding SAM domain are needed to confirm this hypothesis.

### EphB6‐specific amino acid divergence in the nucleotide‐binding pocket

**Fig. 4:**
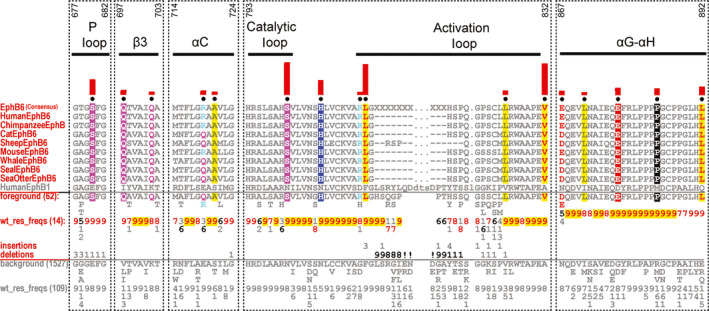
Analysis of EphB6 pseudokinase domain consensus sequences. CHA 31, with selected EphB6 sequence in the foreground and EphB6 sequences from other Eph tyrosine kinase family members in the background alignment. For further details, see the legend to Fig. 1. Regions with strong evolutionary constraints are shown. The amino acid numbering in these regions (shown above the alignment) is based on the human EphB6 sequence. The human EphB1 sequence is provided for comparison. UniProt accession numbers for the sequence analysis are as follows: Human EphB6: O15197; Chimpanzee EphB6: H2QVJ1; Cat EphB6: M3WLM1; Sheep EphB6: W5Q5J1; Mouse EphB6: O08644; Whale EphB6: A0A384ABL8; Seal EphB6: A0A2U3YIR2; Sea Otter EphB6: A0A2Y9JXM3. Human EphB1: P54762.

**Fig. 5:**
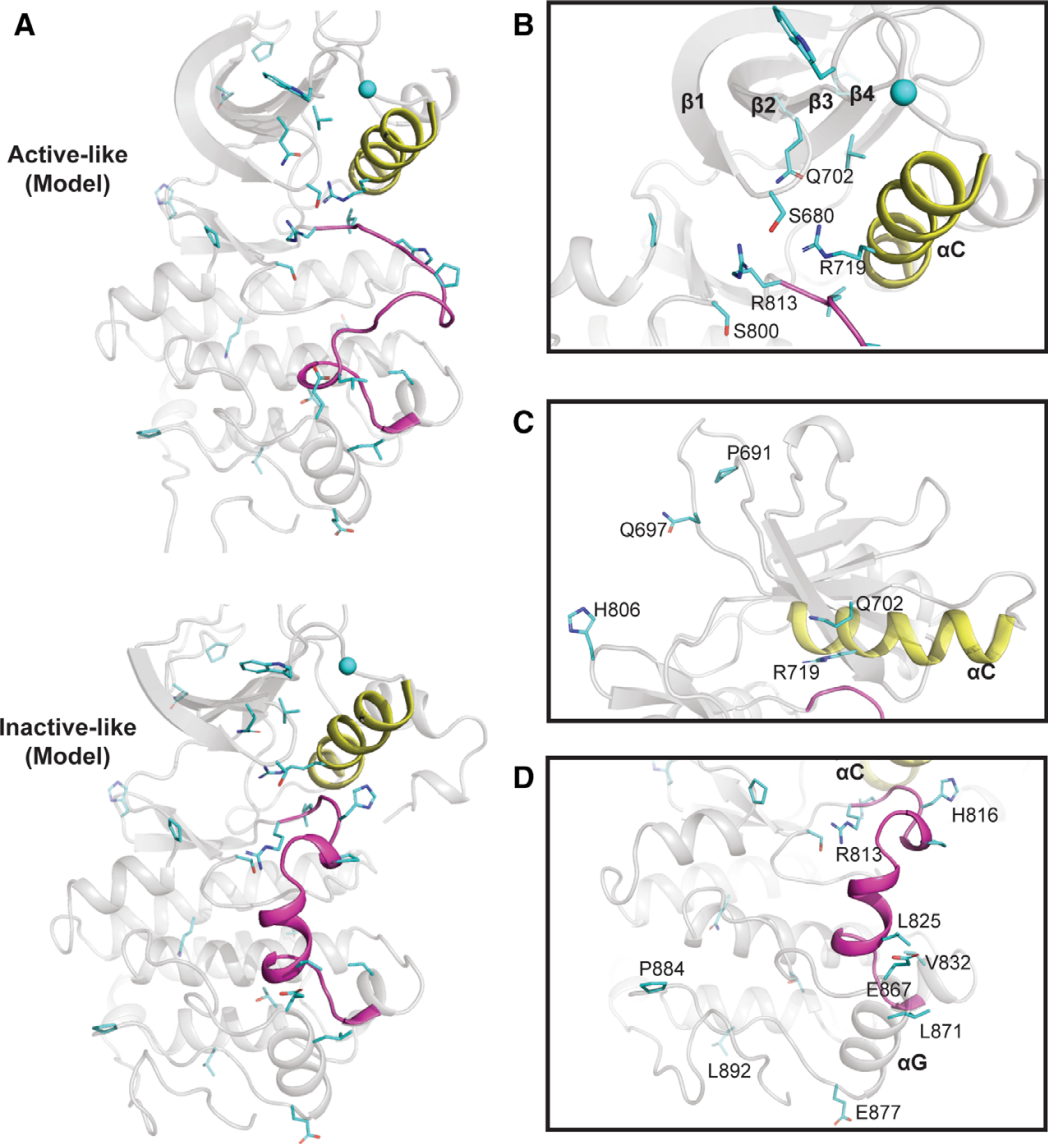
Homology models of EphB6 in ‘active‐like’ and ‘inactive‐like’ conformations. (A) Cartoon representation of the homology models of human EphB6 pseudokinase domain in ‘active‐like’ EphA3 conformation (PDB ID: 3FXX) 39 and ‘inactive‐like’ unphosphorylated apo EphA3 (PDB ID: 2QO2) 36 conformations built using the SWISS‐MODEL server 84. (B) EphB6 divergence in the ‘nucleotide‐binding’ pocket. (C) EphB6‐specific divergence in the N‐lobe of the pseudokinase domain. (D) EphB6‐specific divergence in the activation loop and ‘substrate‐binding’ regions. In A–D, the αC‐helix and activation loop are coloured in yellow and magenta, respectively. EphB6‐specific residues are coloured in cyan and shown as sticks. The residues were identified using the Bayesian procedure described in Fig. 1.

As discussed for EphA10, a quantitative analysis of EphB6 amino acid composition also reveals a number of changes in the putative ATP‐binding pocket that are likely to contribute to unique conformations linked to specific signalling properties of the pseudokinase domain (Table 1 and Fig. 4). In contrast to EphA10, EphB6 has previously been shown to bind to nucleotides *in vitro* and in competition‐based cellular assays employing panels of protein kinase inhibitors. The molecular basis of these unexpected experimental findings is supported from both a sequence (Fig. 4) and modelling analyses (Figs 3 and 5). For example, there is an EphB6‐specific Ser680 adjacent to the second Gly in the Gly‐rich loop instead of the negatively charged Glu residue found in other Eph family members (except for the EphA10 family which has a positively charged Arg, as described above, Fig. 4). By virtue of its small size, a Ser side chain might aid in stabilizing the negatively charged phosphate groups of the ATP nucleotide, due to its ability to form hydrogen bonds, whilst providing a receptive environment for biochemical docking. Interestingly, the ‘invariant’ β3 Lys is replaced by Gln702 in all EphB6 pseudokinases when compared to the canonical EphB1 kinase (Fig. 4). Although the formal positive charge of Lys is lost, Gln can still form hydrogen bonds and may therefore be involved in interaction with ATP in a noncanonical mode that might help trigger conformational switching between ‘active‐like’ and ‘inactive‐like’ conformations (Fig. 5). Interestingly, the αC‐helix glutamate is replaced by a positively charged arginine (Arg719). Moreover, the magnesium‐binding Asp is also a positively charged arginine residue (Arg813) in the EphB6 family and the second magnesium‐binding Asn is replaced by a serine (Ser800). Together, the co‐evolving residues specific to EphB6 in the ATP‐binding pocket (Figs 4 and 5B) predict a metal‐independent mode of ATP binding, because negatively charged residues that target divalent cations are replaced with either positively charged side chains or uncharged residues capable of mediating hydrogen bonds (Fig. 3). The lack of metal‐binding motifs, coupled with the conservation of Arg813, explains why EphB6 can bind to nucleotides independently of any cations 28 and is worthy of further investigation if pharmacological inhibition of EphB6 is pursued in the future therapeutically.

### EphB6‐specific divergence in the substrate‐binding pocket

By definition, the substrate‐binding pocket of pseudokinases is not generally thought to support canonical protein phosphorylation *per se*. However, excitingly, atypical mechanisms of catalysis have been described in several pseudokinases 42, 43, whilst atypical kinases such as the WNK subfamily utilize conventional amino acids at unconventional positions to support catalysis 44. These findings hint at a currently untapped wealth of protein kinase biochemistry that awaits future discovery. Moreover, new kinase‐independent regulatory functions can emerge in pseudokinases, driven either through nucleotide or through regulated protein:protein binding interactions, and although EphB6 and EphA10 are not believed to form a heterodimer 45, there are several lines of evidence demonstrating productive heterodimersation complex formation between Eph pseudokinases and canonical Eph kinases 4, 45, 46, 47, 48. The EphB6 activation segment is highly degraded (Table 1), suggestive of the loss (or gain) of a functional role for amino acids in the short activation loop. For example, His816 could act as an interaction interface between the JX domain and αC‐helix because of its unique ability to form two hydrogen bonds through its side chain. In addition, Pro691, Asn697 and His806 are located in loops on the same face of the EphB6 pseudokinase domain (Fig. 5C). We therefore predict that they could together form an interaction site for binding of partner proteins. We also found specific, conserved, amino acid changes in the αG‐helix, which might permit use of this region as a docking site(s) for protein:protein interactions. Specifically, Glu867 and Leu871, together with Leu825 and Val832, could form a unique binding site with other (catalytically active?) members of the Eph family (Fig. 5D). Moreover, Glu877 and Pro884 in the αG‐αH loop and Leu892 in the αH‐helix could tether the SAM domain linker in a unique conformation and, thus, stabilize this domain in regulated orientations that are capable of driving appropriate oligomerization patterns.

### Tribbles and STK40 pseudokinases: silent but deadly

**Fig. 6:**
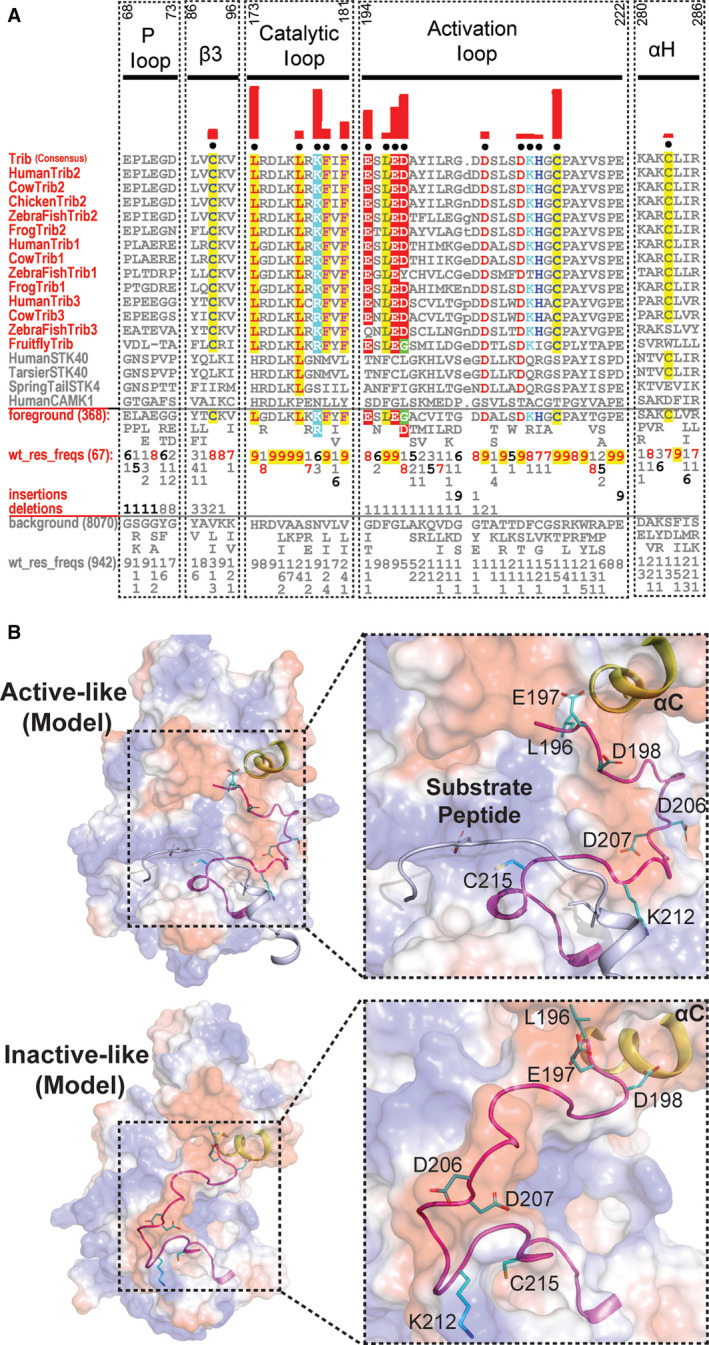
Analysis of unique features of TRIB and STK40 pseudokinases in the activation segment. (A) CHA 31, showing sequence constraints distinguishing TRIB kinases from other closely related CAMKs. Selective TRIB sequences from diverse organisms are shown in the display alignment. Foreground sequences include TRIB pseudokinases, whilst the background includes related CAMK sequences; human canonical CAMK1 is provided for reference. Please refer to Fig. 1 legends for further details. (B) Surface electrostatic of TRIB2 homology models in the ‘active‐like’ (PDB ID: 6DC0) and ‘inactive‐like’ (PDB ID: 5CEM) 57 states. The activation segment of the ‘inactive‐like’ state was not resolved in the crystal structure and was therefore modelled by loop modelling in Rosetta 86, 87. Distinguishing pattern residues in the activation loop are shown as sticks. TRIB‐specific residues are coloured in cyan. The C‐helix is coloured in yellow, and the activation loop is shown in magenta. Substrate peptide in the left panel is shown as a cartoon and coloured in blue/white. UniProt accession numbers for the sequence analysis are as follows: Human TRIB2: Q92519; Cow TRIB2: Q5GLH2; Chicken TRIB2: Q7ZZY2; Zebrafish TRIB2: E7F3S2; Frog TRIB2: Q76D08. Human TRIB1: Q96RU8; Cow TRIB1: A6QLF4; Zebrafish TRIB1: E7FD70; Frog TRIB1: F7BWB1. Human STK40: Q8N2I9; Tarsier STK40: A0A3Q0E403; Springtail STK40: A0A1D2MVE9. Human CAMK1: Q14012.

Tribbles 1‐3 and Sgk495/serine/threonine kinase 40 (STK40) form a small four‐member subfamily of human pseudokinases, which are most closely related to the fly pseudokinase termed TRIB 49. TRIBs all contain an atypical catalytic site (Table 1 and Fig. 6) that co‐evolved with a small motif in the C terminus that controls the stability of CDC25 50 and regulates the ubiquitin‐driven turnover of the tumour‐associated transcription factor CCATT‐enhancer‐binding protein (C/EBP)α 51. More broadly, TRIB1, TRIB2 and TRIB3 contain additional conserved sequence motifs that permit them to engage and tune different aspects of canonical MAPK and AKT‐based signalling 49. Consistently, atypical TRIB expression patterns are linked to cancer pathology, inflammation, neurological disorders and metabolic regulation in humans. Cellular overexpression of TRIB2, the most ancestral of the TRIBs, has been most strongly associated with different human cancer subtypes, including drug‐resistant malignant melanoma 52. In addition, TRIB2 regulates the WNT, YAP and C/EBPα pathways in hepatic cancer 53 and C/EBPα in a model of non‐small‐cell lung cancer 54, as well as possessing complex oncogenic/tumour‐suppressive outputs in the aetiology of AML and ALL 55, 56. The TRIB1 pseudokinase functions as a dynamic signalling scaffold that recruits substrates to be ubiquitinated as part of a pseudokinase/E3/pseudosubstrate ternary complex 57. Like TRIB1, TRIB2 represents a still poorly characterized mediator of proliferative signalling pathways, and TRIB2 modulators (such as conformation‐biased inhibitors) may be good therapeutic agents in multiple cancers especially in the context of drug resistance. Recent work demonstrates that TRIB1 46 and TRIB2 47, and by extension TRIB3 and STK40, are targets of chemotypes represented in commercial kinase inhibitor libraries and clinical compounds 46, 47, 58, 59. The finding that TRIB2, but not TRIB1 or STK40, possesses an ability to bind (albeit weakly) to ATP and also autophosphorylate very weakly *in vitro* in a metal ion‐independent fashion 60 is consistent with the unusual TRIB2 nucleotide‐binding site (Table 1), which lacks canonical amino acids for binding to metals, as discussed above in the context of EphA10 and EphB6. However, like EphB6, the atypical nucleotide‐binding site and regulatory regions are available for small molecule targeting. Human TRIBs and STK40 were originally identified as pseudokinase homologues of the fly TRIB protein. However, despite accumulating data attesting to the importance of STK40 in (patho)physiologic processes 61, 62, little is known about its biological regulation or intracellular mechanism of action. Like TRIB proteins, STK40 interacts with constitutive photomorphogenic protein 1, relying primarily on a C‐terminal sequence that is highly analogous to the auto‐associating tail motif found in human TRIB proteins 61, 63. As demonstrated for TRIB1, substitutions of conserved residues within the STK40 pseudokinase domain prevent ATP binding, confirming that STK40 is a catalytically inactive pseudokinase 63.

### Allosteric transitions in modelled Tribbles and STK40 pseudokinases

The vertebrate TRIB pseudokinases contain three separate regions, an unstructured N‐terminal domain rich in PEST sequences, a central conserved pseudokinase domain and a C‐tail, which contains a highly conserved peptide sequence that docks into the atypical nucleotide‐binding site 49. TRIBs share co‐conserved patterns in the catalytic domain (Table 1), which distinguishes them from all other protein kinases and also formally separates TRIBs from STK40/SgK495 and canonical Ser/Thr kinases such as calcium calmodulin kinase (CAMK)1 (Fig. 6A). TRIBs exhibit shared high sequence identity in the pseudokinase domain: human TRIB2 is 71% identical to TRIB1, whereas TRIB3 possesses 54% identical amino acids. Although TRIB pseudokinases are the closest relatives of STK40/SgK495, sequence identity is highest for TRIB3 (21%), but similar to TRIB1 (20%) and TRIB2 (19%). Of evolutionary interest, the TRIB1 crystal structure demonstrates that the SLE residues that replace the canonical ‘DFG’ motif permit specific adaptions to stabilize the activation loop in a unique ‘inactive‐like’ conformation that precludes ‘substrate’ binding. Since TRIBs provide a regulated scaffold to target ubiquitin E3 ligases, these ubiquitin transfer enzymes can formally be considered as ‘substrates’, although there is no evidence for cellular substrate phosphorylation by TRIB pseudokinases in the canonical sense. Upon peptide substrate binding, or *in cis* interactions with the C‐tail or C/EBPα 49, the SLE motif undergoes coordinated conformational toggling, interconverting TRIB1 between an ‘inactive’ conformation in which the activation loop blocks the pseudoactive site (also termed ‘SLE‐out’) and a more open substrate‐bound (also termed ‘SLE‐in’) conformer (Fig. 6B), whilst simultaneously reshaping the ‘substrate’‐binding site. This substrate‐bound ‘SLE‐in’ conformation in TRIB1 resembles the ‘DFG‐in’ conformation of canonical kinases, consistent with positional replacement of the ‘DFG’ metal‐coordinating motif with the SLE sequence in TRIB pseudokinases 46. Based on our informatic analysis, we predict a similar mechanism of transitional conformational regulation for both TRIB2 (Fig. 6A,B) and TRIB3, whilst STK40 diverges somewhat in various TRIB‐conserved positions 49. However, like one structural snapshot of TRIB1 and those modelled for TRIB2 (Fig. 6),the STK40 pseudokinase domain 63 crystallizes in an ‘active‐like’ closed kinase fold, despite failing to bind nucleotides and remaining catalytically inactive. Interestingly, like the TRIBs, the STK40 catalytic loop contains the XRD sequence found in catalytically active kinases (typically HRDxKxxN). Hydrophobic residues corresponding to the regulatory spine (RS) positions of active kinases (which are also found in TRIBs, Table 1) make packing interactions that stabilize conformational interactions between the N‐ and C‐lobes of STK40 that permit adoption of an ‘active‐like’ conformation, which possesses marked similarity in the N‐lobe to the active PKA structure 63. However, like EphA10, EphB6 and TRIB1, STK40 lacks key motifs required for catalytic activity, in addition to a highly degraded Gly‐rich loop that does not permit ATP binding (Table 1). This region is divergent from canonical kinases and both TRIB and Eph kinases and pseudokinases, with two of the canonical glycine residues replaced by a serine and a proline (Gly‐X‐Ser‐X‐X‐Pro). In addition, STK40 contains substitutions in the β2 (Val) and β3 (Val‐Ala‐x‐Lys) regions, and the Ala in β3 is substituted at Gln64. The size of the amino acid at this position has been suggested to be diagnostic for deficiency in ATP binding in some (pseudo)kinase domains 41, 63, although Ala is found in both EphA10 and EphB6 ‘VAIK’ motifs, which is consistent with an ability to bind to ATP (Table 1).

### Are TRIB pseudokinase outputs tuned through regulatory Cys‐based mechanisms?

Analogous to conventional kinases, ‘substrate’ interactions are thought to induce conformational changes in the flexible (but highly degraded) C‐helix and activation segments of both TRIB1 46 and TRIB2 (Fig. 6B). However, TRIB are unusual, even amongst pseudokinases, due to the conservation from flies to humans of three specific invariant Cys residues. The first is found in the β3 strand motif, where a conserved Cys (Cys89 in TRIB2) precedes the canonical ATP‐binding Lys residue (Lys90, Fig. 6A). Vertebrate TRIB pseudokinases also contain a conserved Cys in the αH‐helix (Cys283) and a conserved Cys that lies in the equivalent position of the T‐loop site of phosphorylation in canonical kinases (Fig. 6A, Table 1). In active kinases, conformational changes are associated with a switch between low and high catalytic states of activity that are also relevant to drug binding 64, 65, 66, 67. This switch can be driven by (de)phosphorylation cycles within the activation loop 68. How, or if, the TRIB activation loop conformation is regulated by post‐translational modifications is unknown, although biophysical evidence suggests that intramolecular interactions between the C‐tail region of TRIB1 and TRIB2 can convert the pseudokinase from an ‘inactive’ open form to an ‘active’ closed signalling platform, driven by C‐helix dynamics that are coupled to the atypical activation segment 46, 58. Sequence analysis (Fig. 6A) suggests that by analogy with canonical kinases, the TRIB kinase activation loop might also be regulated. Apart from a few Ser residues, there is a general lack of highly conserved phosphorylatable residues in the classical T‐loop region, and conserved Asp residues appear to dominate instead (Table 2, and see below). However, a highly conserved Tyr residue in the P + 1 loop of vertebrate TRIBs (Tyr218 in human TRIB2, Fig. 6A) might conceivably fulfil a phosphodependent role, as recognized in the control of substrate binding in canonical kinases such as Mps1/TTK 69, 70, PLK1 71 and PLK4 65. However, our analysis of the evolutionary constraints acting on TRIB amino acids clearly reveals a distinctive Cys residue (Cys215 in TRIB2) in the activation loop in all extant TRIB (but not STK40) pseudokinases, which raises the possibility of dimerization and/or redox control of TRIB kinase conformations (Fig. 6A,B) and protein:protein interactions. The residue equivalent to Cys215 in TRIB2 is usually conserved as a Ser or Thr in non‐TRIB kinases (including STK40), permitting phosphorylation‐driven conformational switching, which is especially well‐understood for ‘RD’ kinases, in which an Arg residue in the catalytic loop is involved in binding the phosphorylated T‐loop residue 68, although this Ser or Thr residue lies between three and five amino acids N‐terminal to Cys215. Interestingly, an Arg is still conserved as part of the ‘atypical’ RD motif in TRIB2, TRIB3 and STK40 (Table 1), whilst Cys remains one of the highly conserved amino acids in the activation segment. The reactive (nucleophilic) thiol group of Cys can partake in a versatile set of chemical reactions, including transiently and stably oxidized species, nitration and phosphorylation 72 and switchable disulfide‐driven complexes that create new opportunities for regulated higher‐order protein assemblies. Cys also has the ability to coordinate metal ions, which, in the context of protein kinases, might play important roles in nucleotide‐dependent catalysis, ion sensing and/or the binding of other proteins, including pseudosubstrates positioned for ubiquitylation (in the case of TRIB pseudokinases) by the ubiquitin E1/E2/E3 Cys‐based catalytic system. Interestingly, recent work demonstrates that the unusually high conservation of Cys residues in TRIB2 can be exploited serendipitously for low‐affinity drug targeting with clinical electrophilic inhibitors such as afatinib and neratinib 47. Cys‐dependent conformationally tractable effects on TRIB2 are evident *in vitro* and cells, where they probably drive interconversion of ‘inactive‐like’ and ‘active‐like’ TRIB2 conformations (Fig. 6B) similar to those described for TRIB1 46, 57.

### Acidic TRIB‐specific activation loop residues important to pseudosubstrate binding?

Two invariant residues conserved in the activation segment, Asp206 and Asp207, might also potentially change the electrostatic surface of TRIB pseudokinases to enable ‘substrate’ binding (Fig. 6A,B). Interestingly, a positively charged groove in the TRIB substrate‐binding pocket is occluded by the negatively charged aspartates in the ‘inactive‐like’ state (Fig. 6B). In terms of regulation, a large positively charged groove is created in the substrate‐binding pocket when the pseudokinase is in the ‘active‐like’ state (Fig. 6B). The relevance of these acidic groups to TRIB‐based cellular signalling is worthy of further investigation.

### PSKH1 and PSKH2 (protein serine histone kinases 1 and 2): The ‘darkest’ of kinases?

**Fig. 7:**
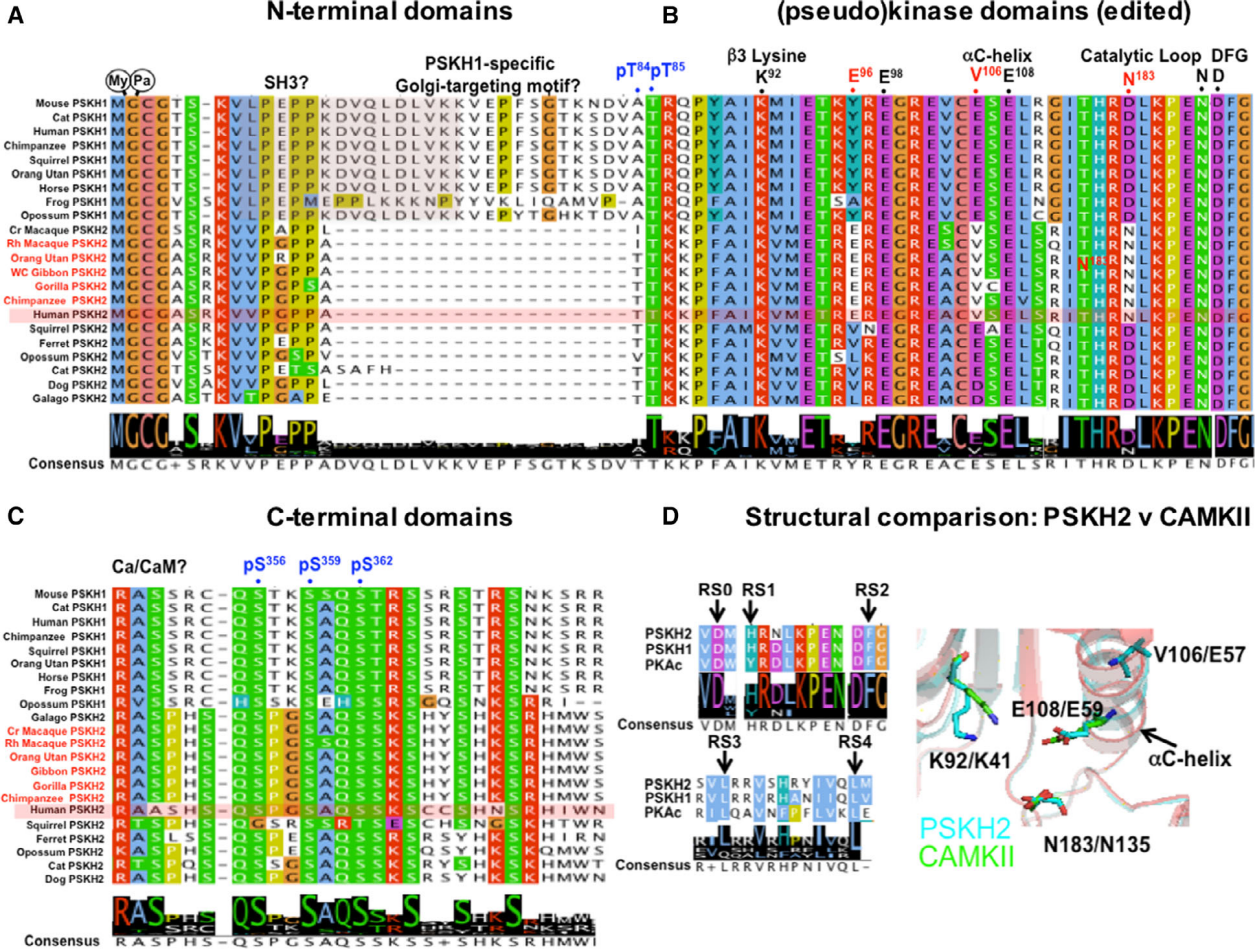
Comparative analysis of vertebrate PSKH1 and PSKH2*.* Amino acid alignment of selective conserved and unique regulatory sites and catalytic motifs (above, black) comparing human and other primate PSKH2 orthologues highlighted (red) with PSKH1 and nonprimate PSKH2. (A) PSKH1 and PSKH2 N termini from 22 vertebrate genomes aligned in Jalview 88, with human PSKH2 shaded. (B) Selected catalytic amino acids/motifs aligned (conserved primate PSKH2 changes in red, human numbering), with potential human PSKH2 phosphorylation sites shown in blue. Primate‐specific hot spots of amino acid identity in PSKH2 orthologues are evident. (C) Divergent PSKH1 and PSKH2 C termini, with speculative human phosphorylation sites shown in blue. (D) Five classical putative RS residues found in PKA catalytic domain 89 are also conserved in PSKH1 and PSKH2 (arrows), suggesting that an ‘active‐like’ fold is possible in both. PSKH2 (cyan sticks) exhibits catalytic potential based upon HHPred model using CAMKII (green sticks), the closest kinase at the amino acid level for which a structure is available. The predicted PSKH2 αC‐helix catalytic residue Glu108 might interact with the β3 Lys92, equivalent to the Glu59:Lys41 interaction formed in active Ser/Thr kinases. Val106 (notably a Glu side chain in nonprimate PSKH2) also lies on the αC‐helix. Asn183 of PSKH2 adopts a similar conformation to CAMKII Asp135Asn (note that this mutation was introduced experimentally in order to generate a kinase‐inactive mutant for crystallography). This analysis raises the possibility that N183D PSKH2, perhaps alongside other PSKH2‐specific residues found at Glu96 and Val106, might promote catalytic activity in the presence of Ca^2+^/calmodulin.

On the human kinome tree, a distinct ‘dark’ pseudokinase, termed PSKH2, is also most similar to canonical members of the CAMK1/2 arm of the kinome, where it forms a two‐member group of ‘protein serine histone kinases’ 8, 73. The biology of PSKH2 remains obscure, but it is most closely related to the Golgi‐associated canonical kinase PSKH1, which is a catalytically active member of the Ca^2+^‐CAM‐dependent protein kinases 74. Although PSKH1 and PSKH2 share many features in canonical catalytic residues (Table 1), they also possess subtle differences when evaluated side‐by‐side (Fig. 7), most notably a validated Golgi‐targeting motif that is embedded in the N‐terminal region of PSKH1 75 that is conspicuously absent in PSKH2 (Fig. 7A). This makes it unlikely, but theoretically still possible, that putative noncatalytic functions of PSKH2 might be performed by PSKH1 in organisms lacking PSKH2. In contrast, this pseudokinase‐specific region deletion in PSKH2 hints at distinct spatial and membrane identity‐determining roles that are distinct between each of the two proteins, although it is of interest that the putative SH3 binding motif found in PSKH1 is also conserved in PSKH2, as are putative sites of myristoylation and palmitoylation at the N‐terminal second Gly and third Cys positions, respectively (Fig. 7A). Dual acylation of PSKH1 has been shown to be important for Golgi targeting, whilst nonpalmitoylated PSKH1 remains in the ER 75. Although further basic regulatory and substrate‐based details have yet to emerge for PSKH1, the PSKH2 pseudokinase domain remains essentially unstudied, based on the published literature, although changes in PSKH2 levels can be readily assessed through transcriptomic and proteomic approaches 60, 76.

### PSKH2: a model for evolutionary kinase to pseudokinase transitions?

**Fig. 8:**
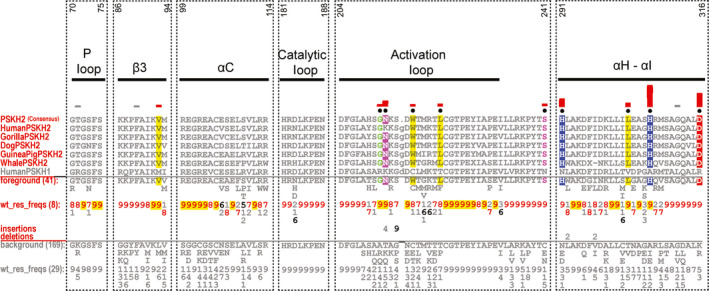
Bioinformatic analysis of the PSKH2 pseudokinase domain. CHA 31 showing the sequence constraints distinguishing selected PSKH2 sequences (foreground) from PSKH1 sequences (background). Display alignment includes PSKH2 sequences from diverse organisms. The number at the top is based on the human PSKH2 sequence (UniProt ID: Q96QS6). Human PSKH1 sequence is provided for comparison. For further details, see the legend to Fig. 1. UniProt accession numbers for the sequence analysis are as follows: Human PSKH2: Q96QS6; Gorilla PSKH2: G3QCY4; Dog PSKH2: F1PBJ9; Guinea pig PSKH2: H0VF24; Whale PSKH2: A0A383ZLZ2. Human PSKH1: P11801.

Remarkably, PSKH2 contains a single amino acid change in the canonical HRD motif, which is converted to HRN in higher chordates (Table 1), analogous to similar changes in the HER3 and Janus kinase (JAK)2 pseudokinase domains 27. This mutation usually abolishes catalysis, since it prevents the Asp acting as a catalytic base to abstract a proton from the OH group in the substrate, but this change has also been found to support catalysis in HER3 77 and JAK2 Tyr‐based pseudokinases 78. This ‘Asp‐to‐Asn’ swap phenomenon, which is observed only in higher vertebrate PSKH2 homologues 49, has escaped scrutiny at the molecular or evolutionary levels, despite its evaluation in human kinome analyses 8 with a hidden Markov model *P* value of 10^−60^, inferring close similarity to active human kinases 8. Interestingly, analysis of 22 PSKH1 and PSKH2 sequences (Figs 7 and 8) confirms that PSKH2 is absent in model mouse and rat genomes, explaining why whole animal genetic knock‐outs/ins are unavailable to the community. Several features in the pseudokinase domain and C‐terminal regions contribute to the unique identity of PSKH2, both as a pseudokinase and as a vertebrate‐specific member of the pseudokinase complement of human cells (Fig. 7A–D). For example, active kinases assemble regulatory (R) and catalytic (C) spine motifs, which interact with the αF‐helix and ATP, controlling conformation and catalytic output. PSKH2 and PSKH1 both contain five amino acids (labelled RS0‐RS4) that are known to be required to create the canonical RS, as well as predicted canonical ATP‐positioning residues, as discussed above. In contrast, the only predicted feature of the PSKH2 pseudokinase domain that inhibits catalytic potential is the loss of the putative catalytic Asp residue in the unusual ‘HRN’ motif. Remarkably, the putative catalytic Asp residue is restored in the HRD motif of nonprimate vertebrate PSKH2s such as dog and guinea pig, but not in large mammals such as whales, where a His residue is present (Fig. 7A).

### A comprehensive bioinformatic analysis of PSKH2

**Fig. 9:**
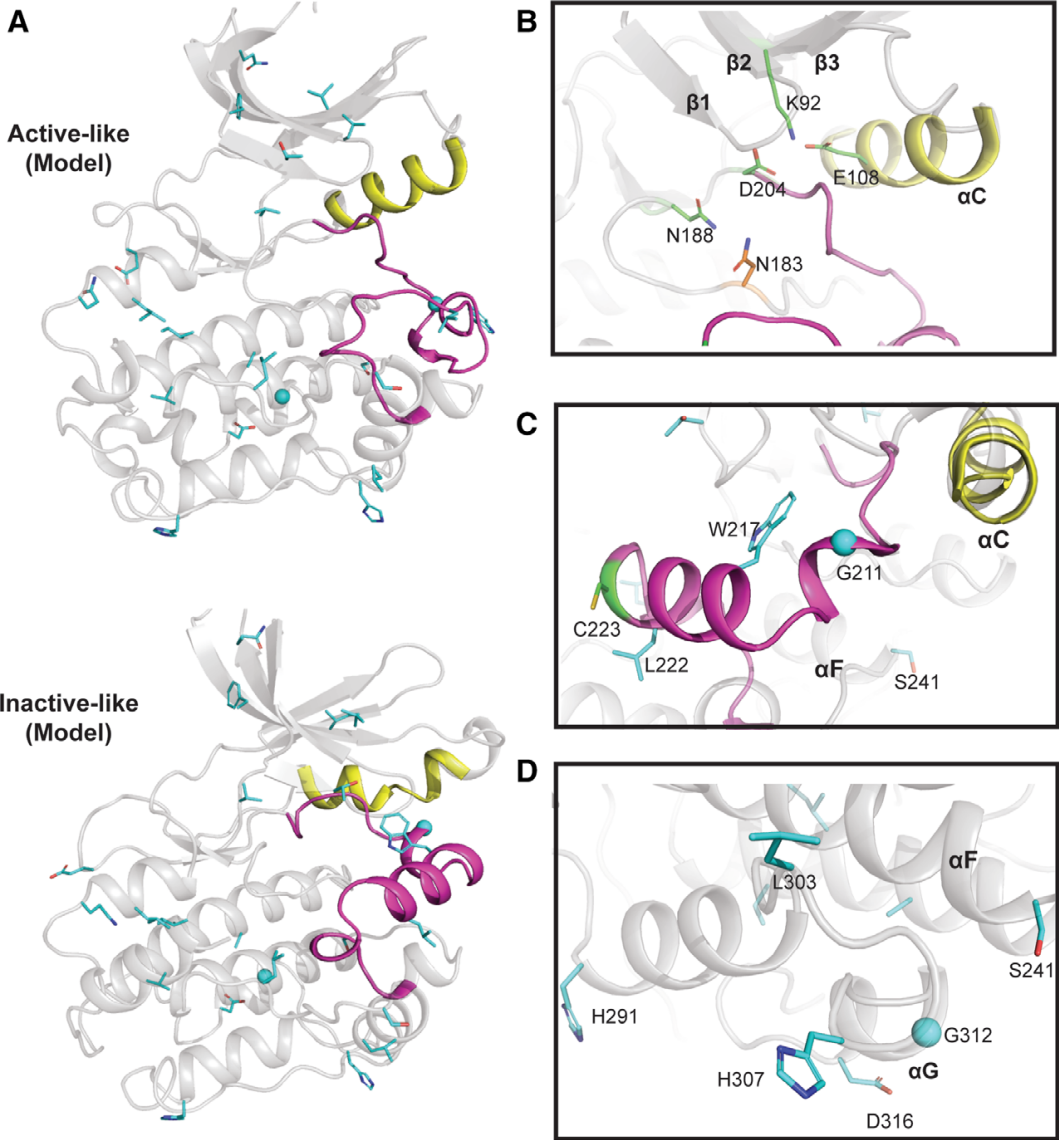
PSKH2 modelled in ‘active‐like’ and ‘inactive‐like’ conformations. (A) Cartoon representation of modelled human PSKH2 in ‘active‐like' (PDB ID: 6BAB) and ‘inactive‐like’ (PDB ID: 4FGB) 90 conformations built using the SWISS‐MODEL server 84. (B) Nucleotide‐binding region of PSKH2. PSKH1‐ and PSKH2‐conserved residues are shown as sticks and coloured in green. Human PSKH2‐specific Asn123 in the catalytic loop is shown in sticks and coloured in orange. Note the potential salt bridge formed between Lys92 and Glu108. (C) PSKH2‐specific divergence in the activation loop and the F‐helix Asp. (D) PSKH2‐specific divergence in the C‐lobe. In A‐D, αC‐helix and activation loop are coloured in yellow and magenta, respectively. PSKH2‐specific residues are shown in cyan sticks. Residues were identified using the Bayesian procedure described in the legend to Fig. 1 (see text for further information).

To begin to dissect PSKH2 functionally, we aligned the complete kinase domain of PSKH2 from multiple species (Figs 8 and S3) and undertook structural modelling of PSKH2 homologues (Fig. 9), in both ‘active‐like’ and ‘inactive‐like’ states present in related canonical CAMKs. This reveals shared and PSKH2‐specific patterns of amino acids in key regulatory regions. Firstly, in terms of quantitative comparisons of PSKH2 with PSKH1 family sequences, several shared features become clear. These include the conservation of the Gly‐rich loop, with a slightly divergent GXGXXS sequence that is, however, predicted to be compatible with ATP binding, a conserved β3 lysine (Lys 92), a C‐helix ion‐pairing glutamate (Glu 108) and magnesium‐binding aspartate (Asp 204) and asparagine (Asn 188) residues, which together predict that PSKH2 is able to bind to ATP in a metal‐dependent manner (Fig. 9B).

### PSKH2‐specific divergence in catalytic motifs, activation segment and the C‐lobe

To help understand any structural basis of PSKH2‐specific adaptions, we created a homology model of PSKH2 using either CAMKII in ‘active‐like’ inhibitor‐bound (PDB ID: 6BAB) or CAMKI in apo ‘inactive‐like’ (PDB ID: 4FGB) states (Fig. 9). The putative catalytic aspartate in the HRD motif is genetically encoded as an asparagine (N183) in human and primate PSKH2 (Fig. 7). However, not all species have an Asn residue at the position. Some conserve Asp or His at the same position, and it is tempting to speculate that deamidation of the Asn residue could also regenerate Asp in those species where it is present. It will be interesting to analyse kinome‐wide proteomic datasets in which variable Asp deamidation is included as a potential modification in relevant kinases and pseudokinases, most notably PSKH2. Alongside the loss of the catalytic Asp, there are two other co‐evolving differences in primate PSKH2, Glu96 and Val106, which are significantly different in PSKH1 and nonprimate PSKH2 (Figs 7 and 8). There are also specific changes in the activation loop of PSKH2. Specifically, introduction of a conserved glycine residue (Gly211) suggests that it might confer a degree of flexibility. In contrast, the introduction of two large hydrophobic activation loop residues, Trp217 and Leu222, could alter activation loop conformation relative to PSKH1, for which structural information is also lacking (Fig. 9C). Analysis of the evolutionary constraints imposed on PSKH2 sequences reveals strong selective constraints on residues in the substrate‐binding C‐lobe (His291, Leu303, His307 and Asp316). Asp316 is especially interesting, as PSKH1 possesses a positively charged (Arg/Lys) residue at this position (Figs 8 and 9D). This could suggest a change in substrate or binding specificity through an electrostatic interaction.

### PSKH2 activation segment and phosphorylatable residues

The region linking β2 and β3 contains potential phosphorylatable residues, including a pair of well‐conserved Thr residues in PSKH2 (Thr84/Thr85) and a Thr in the T‐loop position (Table 2). This Thr residue is common amongst canonical CAMKs, and the additional P + 1 loop Thr and Tyr amino acids are also conserved in PSKH2. A Cys residue is also found two amino acids C‐terminal to the potential phosphorylated Thr in primate PSKH2; this conserved residue is present in most other CAMKs and AGC kinases 79, but is absent in Eph and TRIB pseudokinases. Ser241 is specific to PSKH2, since PSKH1 conserves mostly an Asn, or sometimes Glu or Cys, at this position. Potentially located at the start of the F‐helix, this Ser residue might either represent a site of post‐translational modification or form a large docking interface together with residues His291, Leu303, His307 and Asp316 in the C‐lobe (Fig. 9D). Finally, the C‐terminal region of PSKH2 contains a number of putative conserved phosphorylation sites (Table 2), including a cluster of Ser residues found in PSKH1 and PSKH2 proteins, and a unique Ser residue (Ser348) found only in PSKH2. Several of these residues are reported to be phosphorylated in proteomics databases, but their functional relevance remains unknown.

## Summary remarks and Conclusions

- Pseudokinases are ubiquitous across vertebrate kinomes, where they serve as rate‐limiting dynamic scaffolds and modulators of cell signalling.
- The human Eph receptor superfamily includes the 2 pseudokinase‐containing polypeptides EphA10 and EphB6, the docking of which to nucleotides and intracellular targets might be regulated through conformational flexibility.
- Dynamic TRIB pseudokinase conformations are involved in controlling signalling pathways and are targetable with a variety of small molecules.
- PSKH1 and PSKH2 are a vertebrate kinase:pseudokinase pairing that can be used to probe co‐evolution of amino acids controlling subcellular signalling and help understand their biological roles.

Historically, the study of protein kinases led to the appreciation that precise control of catalytic output (most notably reversible phosphorylation) is important for regulating signalling outputs 6. In this sense, overexpressed or mutated canonical protein kinases have dominated thinking in the field, especially in the context of oncology, where structure:function:druggability initiatives have led to the approval of clinical kinase inhibitors. However, many, if not all, protein kinases also have conventional scaffolding and noncatalytic functions that are likely to contribute, or even dominate, their phenotypic properties, including some that are associated with diseases or targeted by small molecules 4. The recognition of pseudokinases as dynamic scaffolds 80 and/or molecular switches, as opposed to ‘inert’ building blocks, has been important for the development of the field 59, 81, 82, broadening our appreciation of allosteric and scaffolding functions in the whole protein kinase superfamily, and preventing pseudokinase from being neglected as stand‐alone drug targets 12, 13. Pseudokinases within kinomes are by some way the best studied of the multiple pseudoenzyme‐containing enzyme families identified 3, 83, and their molecular analysis in genetically tractable and biochemically manipulated systems has also made them the best understood. However, owing to a relatively low pressure to evaluate biological function outside of a disease context, the analysis of pseudokinases and kinases that make up the ‘dark’ kinome remains patchy. Thankfully, close evolutionary intertwinement within kinomes permits many features of well‐studied members to be evaluated in the context of pseudokinases. In this review, we took advantage of this to compare and contrast three of the least well‐understood pseudokinase subfamilies using sequence‐based modelling. In the case of EphA10 and EphB6, a lack of selection pressure to maintain catalysis (but likely sparing nucleotide binding) is illustrative of heterotypic signalling mechanisms mediated through specific pseudokinase domain adaptations. These might support conformational changes that couple different regions of EphA10 and EphB6 to a range of signalling outputs. Whether these are sensed by ephrin ligand detection, nucleotide binding, or are also a function of stoichiometric complex formation, remains to be established. For Eph pseudokinases, drug targeting of distinct (or a unique) conformations might be added to the wish list of pharmaceutical companies targeting members of the druggable ‘dark’ kinome. Examples include Eph‐dependent signalling in endocrine and immune systems as well as in cancer cell proliferation, where links between EphB6 and EphA10 and canonical kinases such as EphB4, EphB1 and Src‐family kinases have been established. Our analysis of PSKH2 suggests interesting features that distinguish it from PSKH1, but which might also make it a useful model for studying evolutionary and functional constraints that underlie the conversion between canonical kinase PSKH1 and pseudokinase PSKH2. Finally, the pleiotropic TRIB pseudokinases possess unique features, ranging from a key allosteric (druggable) transition through an unusual Cys‐containing activation segment. In this context, an enhanced molecular understanding of the noncatalytic, conformationally driven functions will drive a more thorough understanding of their basic and disease biology.

## Conflict of interest

The authors declare no conflict of interest.

## Author contributions

This manuscript was conceived by NK and PAE, and cowritten by all the authors. The final version was approved by all authors prior to submission.

## Supporting information


**Fig. S1** **.** Comprehensive EphA10 pseudokinase domain sequence analysis.
**Fig. S2** **.** Comprehensive EphB6 pseudokinase domain sequence analysis.
**Fig. S3**. Comprehensive PSKH2 pseudokinase domain sequence analysis.
