# Supplementary material for: Cataloguing the dead: breathing new life into pseudokinase research
Source: FEBS J. 2020 Mar 10;287(19):4150–69. doi: 10.1111/febs.15246 (PMC7586955; doi:10.1111/febs.15246)
Supplement: Supplementary file 1 — Fig. S1 . Comprehensive EphA10 pseudokinase domain sequence analysis. Fig. S2 . Comprehensive EphB6 pseudokinase domain sequence analysis. Fig. S3. Comprehensive PSKH2 pseudokinase domain sequence analysis. [file FEBS-287-4150-s001.zip › febs15246-sup-0001-FigS1-S3.pdf]

FEBS PRESS
science publishing by scientists

WILEY

The FEBS Journal

www.febsjournal.org

Cataloguing the dead: breathing new life into pseudokinase research

Safal Shrestha, Dominic P. Byrne, John A. Harris, Natarajan Kannan and Patrick A. Eyers

DOI: 10.1111/febs.15246

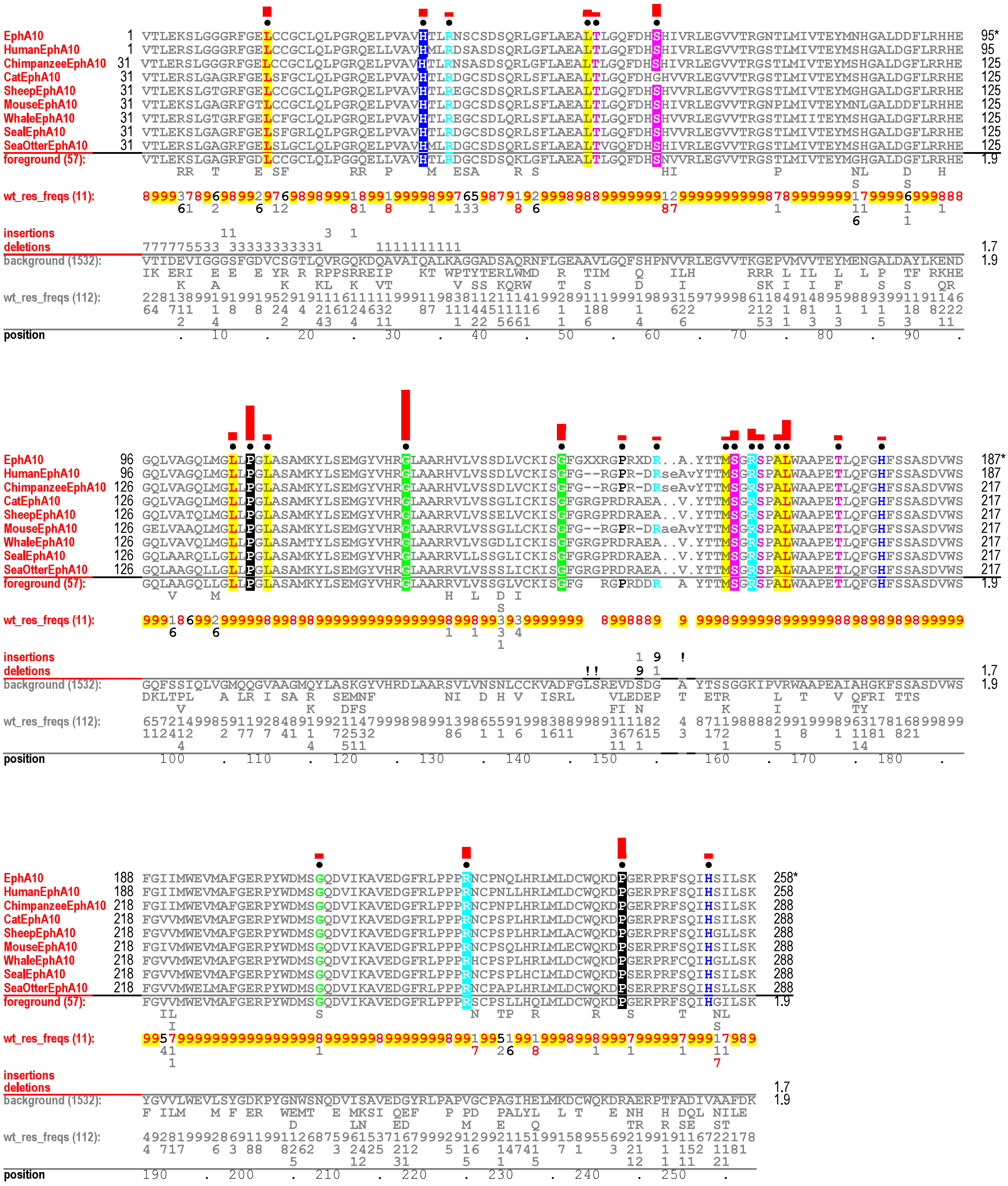

Supplementary Figure S1. Comprehensive EphA10 pseudokinase domain sequence analysis

Sequence constraints that distinguish EphA10 from other Eph sequences are shown as a contrast hierarchical alignment. Complete EphA10 pseudokinase sequences from diverse organisms are part of the display alignment. Numbering at the top is based on the benchmark human sequence, see legend to Figure 1 for more information and [31]. Uniprot accession numbers for the sequence analysis are: Human EphA10: Q5JZY3; Chimpanzee EphA10: H2PYP7; Cat EphA10: A0A212UHA0; Sheep EphA10: W5QF15; Mouse EphA10: Q8BYG9; Whale EphA10: A0A384A623; Seal EphA10: A0A2U3Y845; Sea Otter EphA10: A0A2Y9IWK0.

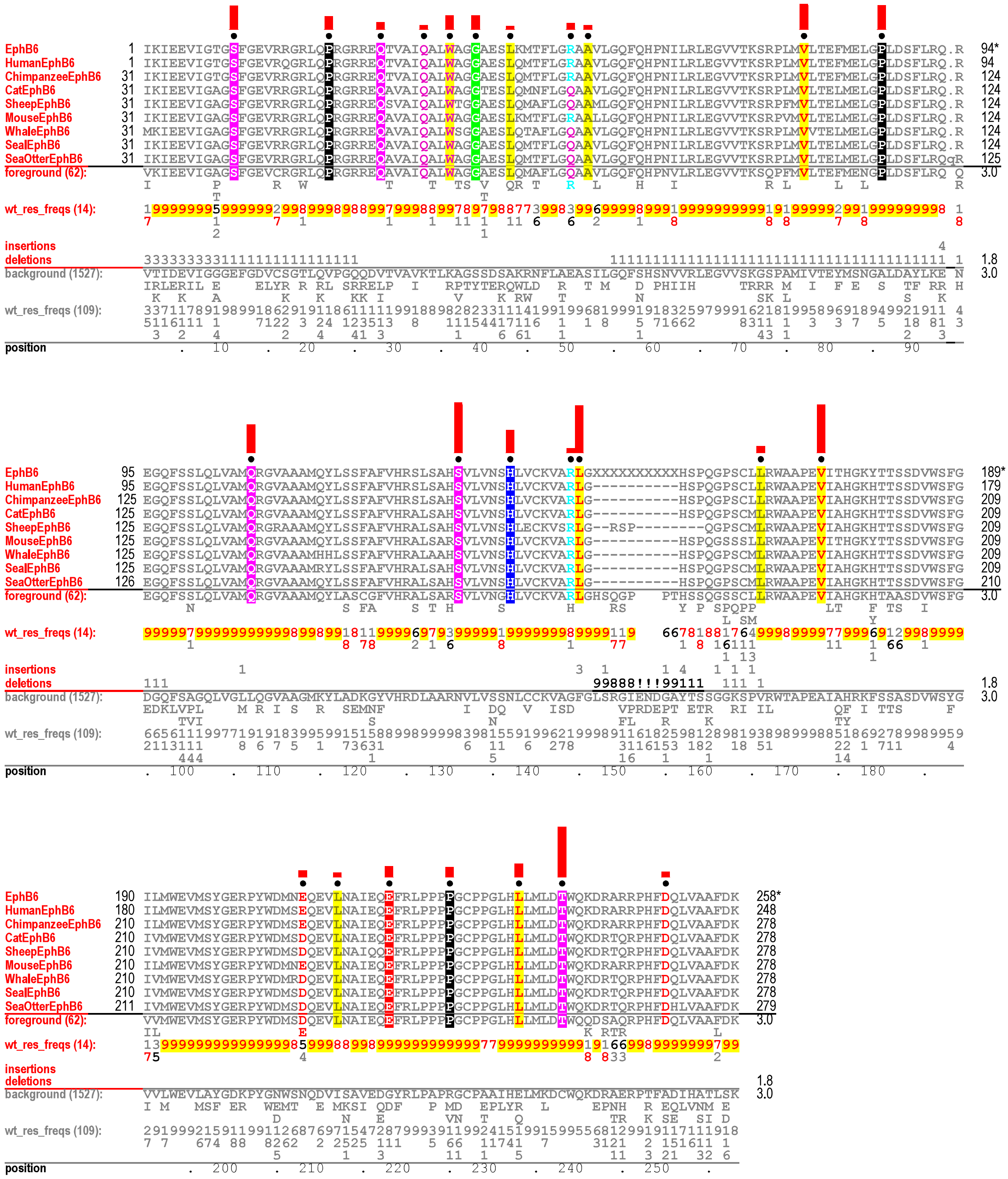

Supplementary Figure S2. Comprehensive EphB6 pseudokinase domain sequence analysis

Sequence constraints that distinguish EphB6 from other Eph sequences are shown as a contrast hierarchical alignment. Complete EphB6 pseudokinase sequences from diverse organisms are part of the display alignment. Numbering at the top is based on the benchmark human sequence, see legend to Figure 5 for more information and [31]. Uniprot accession numbers for the sequence analysis are: Human EphB6: O15197; Chimpanzee EphB6: H2QVJ1; Cat EphB6: M3WLM1; Sheep EphB6: W5Q5J1; Mouse EphB6: O08644; Whale EphB6: A0A384ABL8; Seal EphB6: A0A2U3YIR2; Sea Otter EphB6: A0A2Y9JXM3.

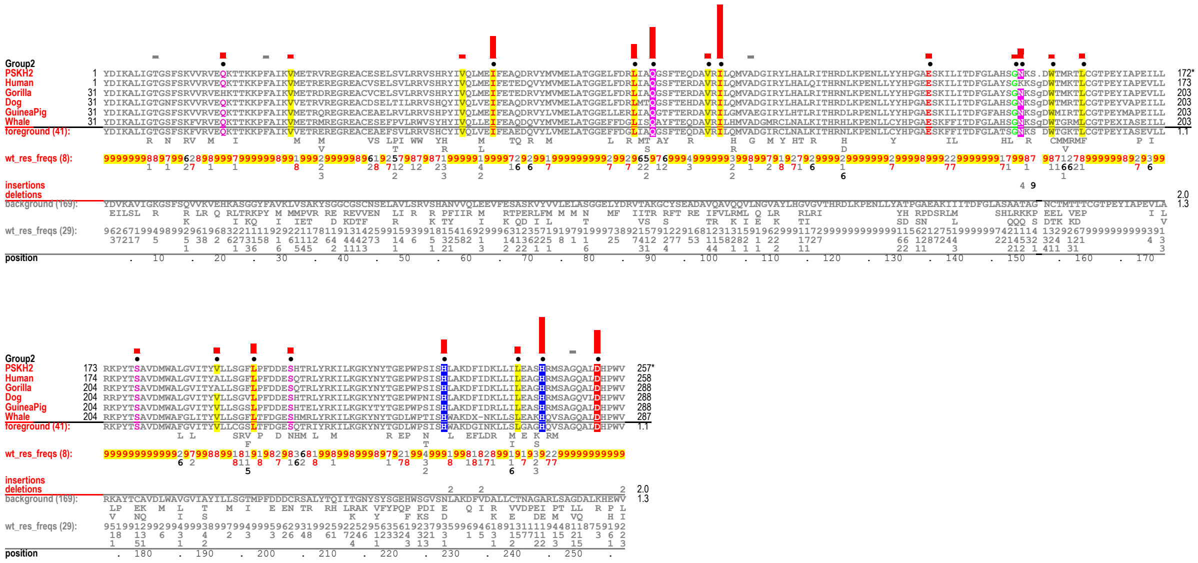

Supplementary Figure S3. Comprehensive PSKH2 pseudokinase domain sequence analysis
Sequence constraints that distinguish PSKH2 from other related kinase and pseudokianse sequences are shown as a contrast hierarchical alignment. Complete PSKH2 pseudokinase sequences from diverse organisms are part of the display alignment. Numbering at the top is based on the benchmarked human PSKH2 sequence. For further information about terminology, please see the legends to Figures 1, 7 and 8 and [31]. Uniprot accession numbers for the sequence analysis are: Human PSKH2: Q96QS6; Gorilla PSKH2: G3QCY4; Dog PSKH2: F1PBJ9; Guinea pig PSKH2: H0VF24; Whale PSKH2: A0A383ZLZ.
